# Photobioreactor design utilizing procedural three-dimensional modelling and ray tracing

**DOI:** 10.1098/rsif.2024.0451

**Published:** 2025-01-29

**Authors:** Kimmo A. Riihiaho, Leevi Lind, Marco L. Calderini, Vilho Halonen, Ilkka Pölönen, Pauliina Salmi

**Affiliations:** ^1^ Faculty of Information Technology, University of Jyväskylä, Jyvaskyla, Finland

**Keywords:** hyperspectral imaging, simulation, light propagation, three-dimensional modelling, microalgae, photobioreactor

## Abstract

The design of photobioreactors for microalgae cultivation aims to achieve an architecture that allows the most efficient photosynthetic growth. The availability of light at wavelengths that are important for photosynthesis is therefore particularly crucial for reactor design. While testing different reactor types in practice is expensive, simulations could effectively limit the range of material and reactor design options. In this study, procedural three-dimensional modelling together with ray tracing was used to create virtual models of a conventional glass photobioreactor lit from the outside and a steel photobioreactor with embedded light sources. The measured transmittance and reflectance of *Chlorella vulgaris* culture were used as a basis for light interaction simulation, and spectral images of the same species were used to validate the simulation results. This type of simulation could have the potential for comparing different reactor architectures, geometries and light attenuation to facilitate the transition to large-scale cultivation. Our results show that the proposed simulator is usable in photobioreactor geometry design as well as in the estimation of available illumination on wavelengths where microalgae have strong absorption peaks, but the handling of light scattering still needs improvement. To the authors’ best knowledge, this is the first attempt, not focused on a specific use case, to build a general photobioreactor design tool capable of estimating hyperspectral light attenuation in microalgae suspension. All software code and used datasets are made available for the reader as open source.

## 1. Introduction

### 1.1. Premise of photobioreactor design

Photobioreactors are containers where biochemical reactions take place in an illuminated environment, often by autotrophic microorganisms [1]. Photobioreactors for the cultivation of autotrophic microalgae are typically designed to be cylindrical, tubular or flat-panel-like, with either an artificial light source or exposure to sunlight [1,2]. Inside photobioreactors, microalgae utilize light energy and carbon dioxide to produce biomass. The biomolecules produced by microalgae, such as pigments and fatty acids, are interesting resources for the biotechnology sector, including the feed industry, cosmetics and nutraceuticals. Despite the acknowledged possibilities for sustainable production, the cost of microalgal production is high, especially in geographic areas where outdoor cultivation is prohibited, or where the electricity and water required for sustaining the cultivation are expensive.

The optimization and design of novel photobioreactors are recognized as an important step in reducing the production costs of microalgae-based goods [1,3,4]. The goal of optimization is to maximize microalgae growth or the production rate at a high biomass level with the least amount of energy input. Maximizing the surface-to-volume ratio of the photobioreactor leads to the highest light availability for autotrophic growth. On the other hand, especially in outdoor conditions, high irradiance and temperature in direct sunlight can inhibit growth [5,6]. Additionally, photobioreactor design should consider aspects such as material costs, lifespan and the possibility of sterilizing, maintaining and controlling the process on an industrially relevant volumetric scale [1]. Photobioreactor design and the optimization of the culturing process for maximal yield of biomass and bioproducts with minimal consumption of resources is a complex problem. In this study, we consider solely light propagation inside a photobioreactor because providing illumination is one of the most energy-intensive aspects of algal cultivation, and it can directly affect the commercial feasibility of a cultivation endeavour [7]. Other important factors in photobioreactor design, such as culturing media, temperature control [7], gas and liquid flow and the optimal shape of a photobioreactor [8], are outside of the scope of this article.

Laboratory experiments of different photobioreactor designs are slow and laborious to conduct, and many parameters, such as light distribution, are difficult to measure in large or complex photobioreactors [9]. Computational fluid dynamics (CFD) modelling is a typical approach to simulate conditions inside a photobioreactor to facilitate their design. CFD models typically consider the geometry of the photobioreactor, the velocity and distribution of the microalgae and the cultivation medium. CFD models have been combined with Beer–Lambertian light transfer and microalgae growth mechanics to estimate the local light environment [3]. In addition to the Beer–Lambert approach, more detailed ray tracing [9] and other Monte Carlo simulations [10] that consider the inherent absorption and scattering properties of microalgae have been proposed to efficiently estimate the propagation of light in cultivation.

Photobioreactors with transparent glass or plastic walls are widely used in laboratories and commercial cultivations. In boreal and arctic conditions, where the cultivations need to be artificially lit, new types of photobioreactors with internal lighting could be designed. In fermentative biotechnology processes, steel cylinders are widely utilized. They are robust, long-lasting and easy to sterilize and maintain. These design features, together with the insertion of light sources inside the steel cylinder-type bioreactor, make them interesting for autotrophic cultivation. Laboratory studies have reported increased microalgae growth when the light source was placed inside a reactor [4].

In addition to the geometry of the photobioreactors, volumetric scale is an important aspect of their architecture [11]. Novel biotechnology processes are typically developed on a laboratory scale, and scaling them up to industrially relevant volumes is difficult. Multiple parameters and configurations need to be considered simultaneously, light being the leading factor for photobioreactors [11]. The problems around photobioreactor design and scaling up laboratory-scale processes to industrially relevant volumes offer an interesting platform for developing new simulation methods. Recently, models based on computer graphics have aroused interest, as they can be used to simulate realistic lighting conditions, including the behaviour of materials, medium and tracing of light propagation on the presented conditions [9]. However, to the best of our knowledge, these techniques have not yet been utilized in photobioreactor design.

**Figure 1.**
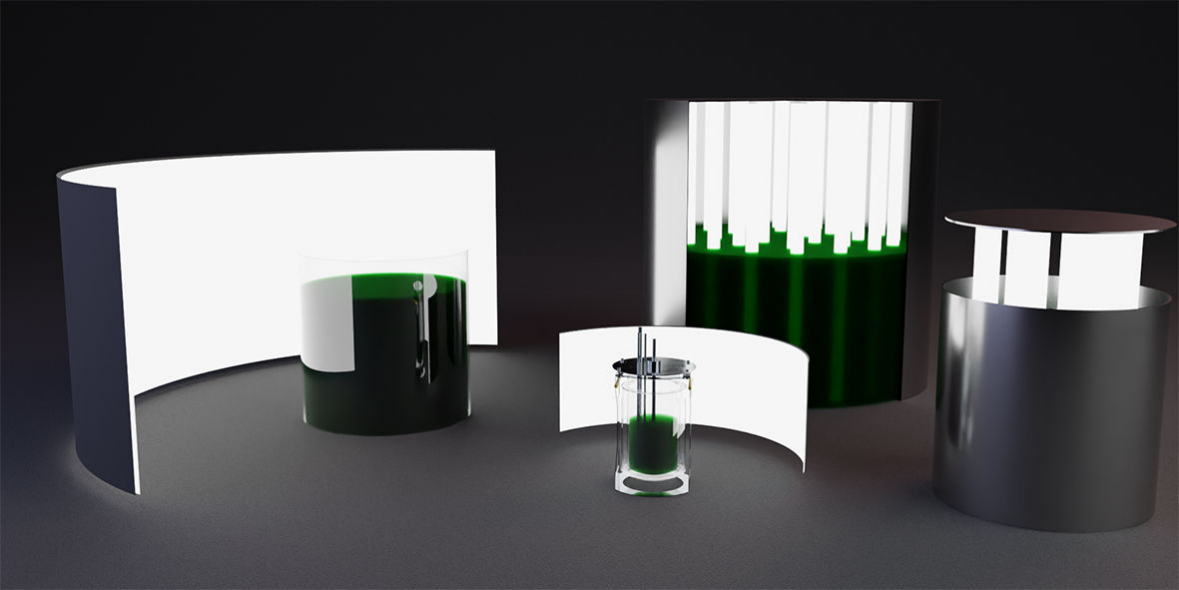
Virtual photobioreactors rendered as an RGB image. Reactor types from left to right: 100 l glass reactor without its lamp, a virtual copy of a 2 l Univessel (Sartorius) reactor, a 1000 l steel reactor with its lid closed and part of the wall cut open and a 100 l steel reactor with raised lid.

The aim of our study was to develop ray tracing and procedural three-dimensional (3D) models of photobioreactors to systematically compare light availability between different photobioreactor materials, geometries and volumes. Our simulation focused on the propagation of light inside a photobioreactor filled with algae suspension of various densities. We simulated a cylindrical glass photobioreactor illuminated from outside of the reactor and a steel cylinder with internal lighting. Both reactor types were simulated as 10 l laboratory-scale units and two industrial prototype scale units (100 and 1000 l). The stylized RGB render of the photobioreactors in figure 1 gives an idea of the virtual materials and the reactor types, but they should not be considered to represent the actual spectral behaviour of the materials and content. The smallest reactor in the centre of the figure is a hand-crafted a virtual copy of a more complicated 2 l Univessel (Sartorius) reactor with mixer blades and aeration tubes that were not used in the experiments of this study. Our simulation allows configuring the light source to match any known spectra to experiment on the penetration capabilities of different combinations of light and microalgae species.

We hypothesized that methods of computer graphics could be used to combine 3D modelling of photobioreactors of defined materials with ray tracing in dense microalgae cultivations. We validated the light field simulations with *in situ* measurements from laboratory cultivation using a hyperspectral imager. This article presents an approach for photobioreactor design that allows feasible scaling of different reactors, the inclusion of materials and the propagation of photosynthetically active wavebands of light in a microalgae cultivation.

### 1.2. HyperBlend

For simulating different photobioreactor designs in a virtual environment, we modified the spectral vegetation simulator HyperBlend [12,13]. HyperBlend has been developed mainly for modelling forest canopies, though the versions published thus far can only simulate the spectral reflectance and transmittance properties of a single leaf as seen by a point spectrometer on wavebands of photosynthetically active radiation (PAR) and near-infrared (NIR).

HyperBlend considers a plant leaf as a volume filled with scattering and absorbing particles that interact with rays of light. Scattering and absorbing properties, as well as the densities of the particles, can be adjusted on a per-wavelength basis. The particles are spread evenly throughout the volume of the virtual leaf, forming a homogeneous mixture [12,13]. A microalgal suspension can readily be modelled as scattering and absorbing particles in a volume as the cells are absorbing and scattering particles themselves.

Adjusting the parameters of the virtual leaf is achieved by adjusting a volumetric shader that defines its optical properties. In computer graphics (CG), a shader is a program that tells light how to interact with a surface or a volume [14,15]. HyperBlend uses the 3D modelling and rendering software Blender [16] as its engine for 3D modelling and rendering tasks. Rendering is a CG term for producing a synthetic image of a virtual world [17]. Blender is not capable of spectral representation of light and can only render images in common RGB (red, green, blue) or greyscale formats. Circumventing this limitation is possible by rendering each wavelength separately as a greyscale image and then combining them into a single hyperspectral image cube [18].

One significant limitation of HyperBlend is that it cannot produce a virtual material accurately if the reflectance and transmittance of the material differ drastically from each other [12,13]. Let us call this an RT-similarity requirement. This does not pose a problem for modelling plant leaves, as their reflectance and transmittance tend to be similar. In general, due to the strongly forward-scattering nature of microalgae in suspension [19], transmittance is much higher than reflectance even in the wavelength regions where microalgae have strong absorption features. This makes the previous HyperBlend version unsuitable for microalgae simulation. To mitigate the problems arising from the RT-similarity requirement and enable the use of HyperBlend in the simulation of light propagation in a microalgae suspension, we retrained HyperBlend’s underlying volumetric shader parameter approximation models, which are covered in detail in §2.2.

### 1.3. Procedural modelling

In 3D modelling, a 3D model is a collection of one or more meshes that define either a bare surface (infinitely thin) or a solid such as a cube, a sphere, or a torus. A mesh consists of a set of corner points called vertices (singular: vertex) that are joined together by edges, which are straight lines between them. Three edges are connected by a surface (called a face) forming a triangle that is the smallest possible unit that can be rendered, as a surface is needed with which light can interact [17, p. 534].

Think of a stool, for example. One could define a cube and four closed cylinders; these would be five separate meshes. If one stuck the cylinders underneath the cube and told the underlying 3D modelling software that these five meshes were now one object, one would get a 3D model representing a stool. Defining a cube is a simple enough task: we have eight corner points and six rectangular faces (split into 12 triangles) that form the solid. However, the cylinders in our example object are more complex shapes to be represented in the discrete world of computers. A cylinder is based on a circle, which is a continuously curving shape. As all computer representations of numbers are discrete by nature, our circle cannot be continuous. In a mathematical sense, even if we use millions of points to define the circumference of the circle, it is still not continuous (though for practical purposes it would be). We must decide how many points are enough. When we begin modelling our stool, we may think that just five points are enough to represent the circumference of a circle for our simple stool model. But what if we want to change this number later?

The traditional way of defining a mesh by vertices, edges and faces is often tedious work. One must define the position of vertices manually and changing the geometry later involves a lot of work whether or not one has modern 3D modelling software at their disposal. In our stool example, adding a single vertex to the circumference would require moving four of the existing vertices along the circumference. If the coordinates of the vertices are set manually, one would have to calculate new coordinates. Often it is easier to just delete the old cylinder and create a new one. However, if one had a more complex shape with lots of work already put into the model, one might be reluctant to start over from scratch.

In this study, we rely on procedural 3D modelling. Procedural modelling means that instead of building the 3D model by adjusting the vertices of a mesh manually, the geometry is generated by a set of rules (e.g. [20]). As an example of this, one could say: ‘Generate a line from $\left(x_{1},y_{1},z_{1}\right)$ to $\left(x_{2},y_{2},z_{2}\right)$ and then run a circle of radius r with n vertices through it’ to form a cylinder. Now if we want to change the number of vertices forming the circumference of the circle, we just give a new n and the mesh is recalculated.

**Figure 2.**
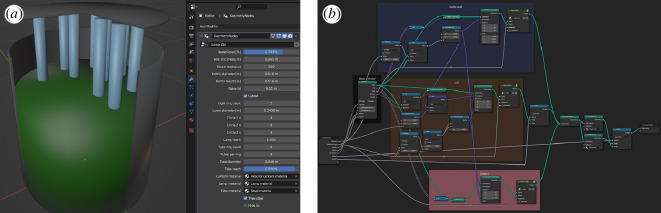
Screencaptures of Blender’s graphical user interface. (*a*) Steel photobioreactor model and its adjustable parameters. (*b*) Node set-up for creating a general adjustable cylinder shell with a lid and a bottom.

In our chosen 3D-modelling tool Blender, procedural modelling is implemented as a visual programming language (VPL) [21] where one connects basic code entities with relations. The code entities are visually represented by blocks called nodes. The entities can be functions ranging from mathematical operations to colour adjustments, or they can represent geometric or mesh primitives used as building blocks for more complex 3D models. The relations between nodes are represented by arcs called noodles. Noodles connect node outputs to other nodes’ inputs. In the Blender community, a program or a subprogram created by its VPL is usually called a node set-up. An example of a node set-up resulting in a solid cylinder shell with top and bottom disks is shown in figure 2*b*. It is a subprogram of the node set-up that generates the whole steel photobioreactor model in figure 2*a*.

## 2. Material and methods

In this section, we cover the cultivation of the microalgae and measure their reflectance and transmittance with a spectrophotometer, modifying the HyperBlend simulator for algal research, constructing a digital twin experiment for validating the simulator, and finally, simulating several different photobioreactor designs.

### 2.1. Spectral measurements of microalgae

*Chlorella vulgaris* (strain CCAP 112/11B) was grown in a 600 ml cultivation flask at 18°C and 48–88 μmol m^-2^ s^-1^ illumination with a 14 : 10 light–dark cycle. The culture was concentrated by centrifugation (3500 relative centrifugal force (RCF), 10 min, 20°C) in 50 ml centrifuge tubes to effectively demonstrate a high biomass cultivation. Cell abundance and biomass of the concentrated sample were measured with an electronic cell counter (CASY, Omni Life Sciences). Measurements were done using a 60 µm capillary (particle size range 1.3–40 µm), and no part of the size distribution was excluded by the evaluation cursors. The cultivation was inspected microscopically to ensure the absence of contaminants. Centrifuged cells were diluted to five concentrations to demonstrate the changes in optical behaviour with varying cultivation density. Cell counts of the diluted samples are listed in table 1.

**Table 1.** Cell counts of the five diluted *Chlorella vulgaris* samples.

sample	cell count (cells ml^−1^)
1	$7.414\text{×}10^{7}$
2	$6.314\text{×}10^{7}$
3	$4.586\text{×}10^{7}$
4	$1.391\text{×}10^{7}$
5	$4.851\text{×}10^{6}$

**Figure 3.**
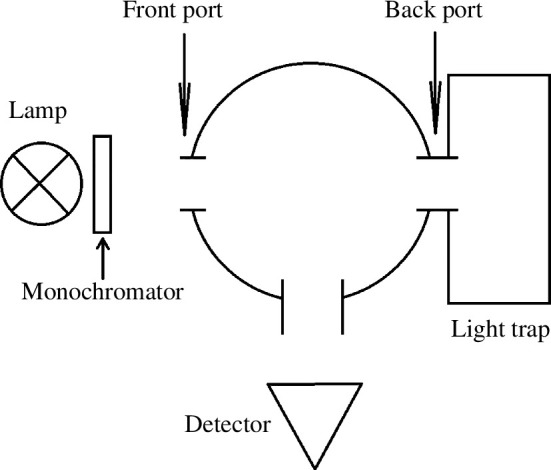
Schematic diagram of the spectrophotometer and integrating sphere set-up used in measuring the spectral reflectance and transmittance of microalgae suspensions.

Transmittance and reflectance of the diluted cultures were measured using a spectrophotometer (Lambda 850 UV/VIS, Perkin Elmer) equipped with an integrating sphere. A glass cuvette (OP38, eCuvettes) with an optical path length of 10 mm and outer dimensions of 50×50×14 mm was used for both transmission and reflection measurements. Transmission was measured by placing the cuvette in the front port of the integrating sphere, and reflection using the back port of the sphere, ensuring that light passing through the sample remained in the light trap behind the integrating sphere. A schematic presentation of the integrating sphere set-up is illustrated in figure 3.

The spectral transmittance and reflectance of the sample were calculated from the measured spectra by removing the effect of the spectrophotometer’s light source. To this end, the spectrophotometer readings were normalized to a measurement made of a white reference sample of Spectralon (Labsphere). The reflection properties of this common reference material are nearly ideally diffusive, with very little spectral variation. A Spectralon plug was placed in the back port of the spectrophotometer while leaving the front port empty. Effectively, this normalization procedure neglects the glass-cultivation and glass–air boundary interactions and thus is a source of systematic error [22]. We assume the error in normalization is negligible compared with the simulation error. Furthermore, in this default measurement set-up, scattered light that cannot enter the integrating sphere port is wrongly attributed to absorption, which overestimates the observable absorption. Scattering can be separated from absorption using an integrating sphere, but the measurement set-up is considerably more complex and prone to errors if not conducted carefully [23]. The simulation properties of HyperBlend are also limited in that it assumes a thin sample and does not consider light that is scattered away.

**Figure 4.**
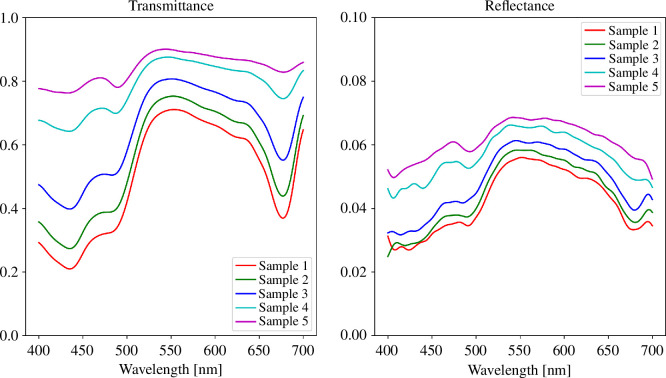
Normalized spectral transmittance and reflectance of *Chlorella vulgaris* in five concentrations. Note the scaling of the *y*-axis in the reflectance plot.

Normalized transmittance and reflectance spectra of the samples are shown in figure 4. The shown spectra are slightly smoothed with SciPy library’s [24] ndimage.gaussian_filer1d method to reduce the measurement noise. These smoothed spectra are also used as the target spectra in the slab simulation to avoid simulating measurement noise. Slab simulation and target spectra are covered in the following section. The transmittance spectra show how the absorption features of the algae strengthen as the cell density increases. The transmittance spectrum of sample 1, with the highest cell density, shows that absorptance in the blue region, from 440 to 500 nm, and in the red region around 675 nm is very strong due to algal pigment absorption. It is worth noting that with increasing cell density, the increase in absorption in the blue is more prominent than the one in the red. In the green region, from 500 to 650 nm, at least 60% of light penetrates the microalgae suspension, regardless of concentration. Spectral reflectance is very low and shows only minimal absorption features throughout the measured range. As expected, the densest sample reflects the least light, and the most dilute sample has the strongest reflectance. Microalgae are known to be strongly forward scattering with roughly 90% of light contained in a 20° solid angle around the forward direction [19], which aligns with our measurements.

### 2.2. Retraining HyperBlend’s neural network approximation

**Figure 5.**
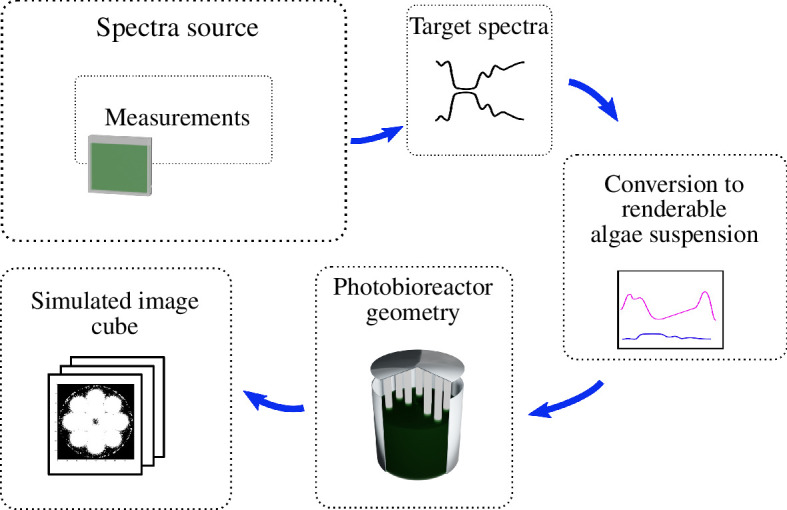
HyperBlend pipeline in photobioreactor simulation.

The complete pipeline of HyperBlend’s spectral simulation is illustrated in figure 5. First, reflectance and transmittance measurements of algae suspension in a glass cuvette are conducted with a spectrophotometer coupled with an integrating sphere to provide the so-called target spectra. HyperBlend takes the target spectra and adjusts the volume shader parameters to replicate that pair of spectra in a virtual cuvette of the same optical path length. This is called a slab simulation: it is the same phase that is called a leaf simulation in the previous forestry-related studies [12,13]. The virtual algae suspension is then enlarged to fill a virtual photobioreactor and rendered into separate greyscale images for each wavelength. These images are finally combined into a spectral image cube.

In HyperBlend’s slab simulation phase (the third step in figure 5), the target spectra are used to adjust the shader parameters so that the virtual material’s spectral behaviour is similar to that of the measured material in the sense of transmission and reflection. This is done one band at a time, so for each band, there is a target reflectance r and a target transmittance t. The slab simulation of the original HyperBlend publication utilized an optimization process to adjust the shader parameters to minimize the difference between the measured and simulated values $\left|r-\hat{r}\right|$ and $\left|t-\hat{t}\right|$, where the simulated values are indicated by a hat [12].

Because the optimization process is computationally demanding and slow to execute, two new methods for estimating the shader parameters were developed in [13]: the surface fitting method and the neural network (NN) method. The core idea behind these methods was simple: first, pairs of reflectance and transmittance values (dubbed RT-pairs) are generated as points in a two-dimensional RT-space, and the optimization process is used to find proper shader parameters for each point. Then one can plot each shader parameter value separately as a function of reflectance and transmittance to form a 3D point cloud that happens to be a nearly continuous surface. For interpolating this surface, the surface fitting model fits an analytical function to the surface and similarly, the NN model fits a neural network to the same surface. Now, each shader parameter can be estimated by a simple function evaluation, or an NN prediction, for any RT pair. This approach yielded significant speed-up in the slab simulation and produced less noisy results [13].

As the optimization method is used for generating the training data points for the surface, and NN method uses a starting guess based on the assumption r=t, the simulation accuracy decreases the further away r deviates from t. In [13], surface and NN methods were trained with data where |r−t|≤0.25, which is sufficient for simulating plant leaves whose reflectance and transmittance spectra tend to have a similar shape and magnitude. For microalgae suspension, which is strongly forward scattering and thus has low reflectance, the simulation must produce accurate results even when t≈1 and r≈0.

In this study, we devised a new iterative training process where the symmetrical starting guess r=t is applied to the first iteration of generated training points as in [13]. The resulting point set was pruned so that each point whose simulation error of either reflectance or transmittance exceeded 2% was excluded from the set. The remaining points were used to fit a surface for each shader parameter to give a new, hopefully better starting guess for the next iteration. As the surfaces to be fitted are smooth by definition, it is reasonable to assume that a small degree of extrapolation can still give a decent starting guess. With this assumption, each following iteration allows r to deviate further away from t and trains a new surface model. Altogether, four iterations were run until the final iteration allowed the extreme situation where either r=1 and t=0 or *vice versa*. The final iteration also trained the NN model using the same procedure and hyperparameters as in [13].

**Figure 6.**
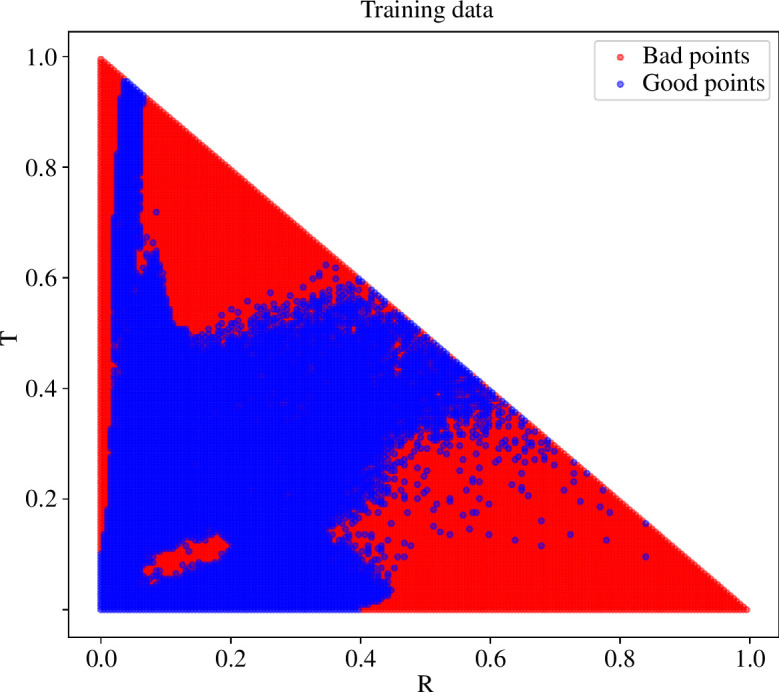
Training data from the last iteration. Points marked as bad exceed the error threshold of 2% either in reflectance or transmittance and were not used in training the final model.

**Figure 7.**
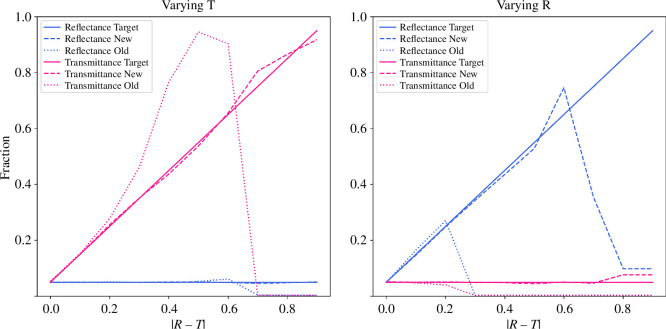
Simulation results contrasted with target values for HyperBlend’s old NN method and the new, iteratively trained, NN method when either r or t is kept constant and letting the other one vary. The simulation results plotted in dashed and dotted lines should stay close to the simulation target plotted as a solid line. The *x*-axis shows the difference between r and t, which is the distance between the blue and the pink target line in *y*-direction. The *y*-axis shows the fraction of transmittance (left) and fraction of reflectance (right).

The iterative training procedure alleviated the RT-similarity requirement considerably, though it was not able to remove it completely. Figure 6 illustrates the generated training data used to train the final models. The RT-similarity requirement is most clearly shown when letting either r or t be a small constant and varying the other one from r=t to 1. In figure 7, this situation and a comparison between the old NN model from [13], and the result of the new iteratively trained NN is illustrated. The old NN breaks immediately when |r−t|>0.2 both on varying r (*right*) and varying t (*left*). The new NN starts to break on varying r at |r−t|≈0.5. Varying t gives fairly accurate results on the whole range. For the sake of the microalgae simulation, the performance in the varying t situation is much more important because, as seen earlier in figure 4, r is low for microalgae suspension while t is high.

### 2.3. Validation experiment

**Figure 8.**
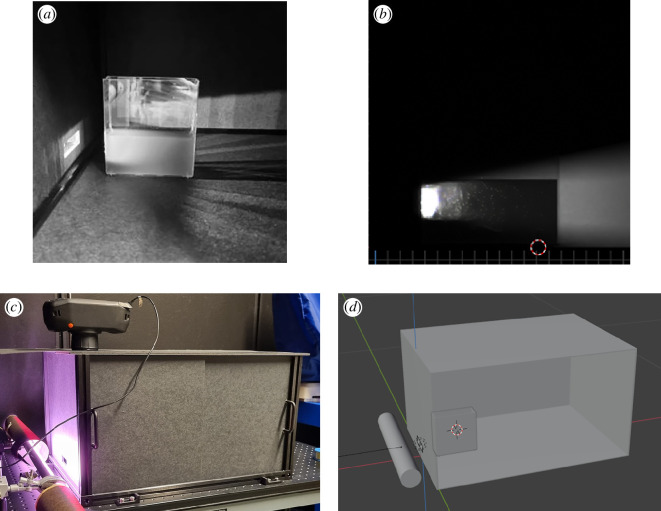
Validation experimental set-up in the laboratory and its digital twin. (a) Microalgae suspension in the open imaging set-up. Red channel of an RGB photograph. (b) Greyscale rendering of the virtual validation scene on 630 nm band. (c) Laboratory imaging set-up. (d) Virtual imaging set-up. The virtual camera is not visible. The front wall of the virtual black box is set to invisible in order to see the cultivation bottle inside for illustrative purposes.

**Figure 9.**
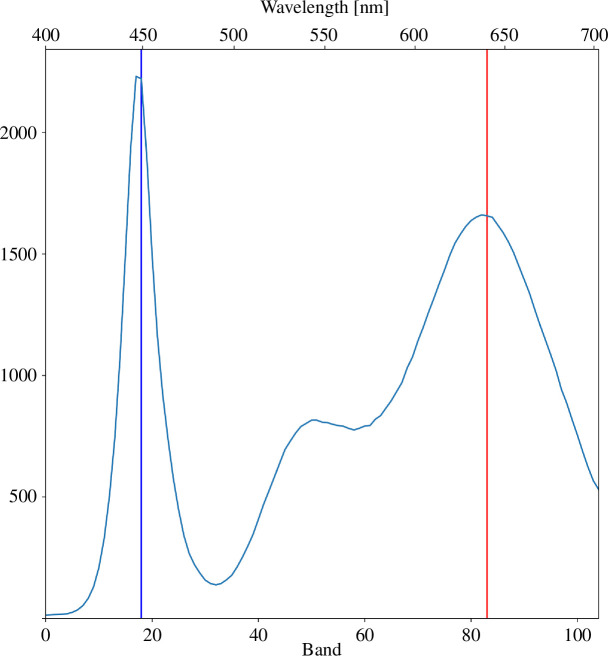
Imaged spectrum of the Valoya AP67 grow light. Vertical lines show the locations of the maximum emission at blue (450 nm) and red (640 nm). The *y*-axis is the digital number recorded by the Specim IQ spectral imager.

To validate that HyperBlend can replicate the most important features of light interaction with microalgae suspension, a digital twin experiment was constructed: a laboratory imaging set-up was replicated in the HyperBlend simulator. Figure 8 shows the laboratory set-up and its virtual counterpart: a microalgae culture was placed in a sawed-off plastic cultivation bottle lit with a grow light (AP67, Valoya), whose spectrum is shown in figure 9. The cultivation bottle was imaged in a black box to avoid reflections from surrounding objects. The light source and the hyperspectral imager (IQ, Specim) were placed outside the box with holes cut into the box to let the light through. The image has 204 spectral bands from 400 to 1000 nm with 7 nm full-width half-maximum (FWHM) spectral resolution. As the photosynthetically active radiation is restricted to the visible region, we utilized only the bands found between 400 and 700 nm in this study. The spatial image size of the imager is 512×512 pixels with a 31° field of view.

Used target spectra were acquired from the spectrophotometer using the same measurement set-up and similarly prepared microalgae culture as those in §2.1. The cell density of the culture was $1.912\;\times \;10^{7}$ cells ml^−1^. The target spectra were interpolated with SciPy’s interpolate.CubicSpline method to 10 nm spectral resolution from 400 to 700 nm, resulting in 31 simulated spectral bands. The root mean squared error (RMSE) of the slab simulation was 1.9% for transmittance and 0.1% for reflectance. The simulated reflectance and transmittance values were compared with the interpolated values. The virtual camera of the simulation was set to a 512×512 pixel image size with a 31° field of view. It is worth noting that the simulated spectral image does not have a meaningful FWHM because each band is discrete and can be thought of as infinitely narrow. The light cone of the laboratory set-up is shown in figure 8*a* as a greyscale image of the red band of an RGB photograph and the light cone of the replicated virtual scene rendered on the brightest band (630 nm) in figure 8*b*. The images are not directly comparable as the red filter of an RGB camera is considerably wider than a single narrow band of a hyperspectral image cube and, thus, the cultivation bottle in the real photograph appears brighter. In addition, there was stray light entering the imaging box from the ceiling lamps of the laboratory when the photograph was taken in figure 8*a*.

As stated in §2.1, both the spectrophotometer measurement and the slab simulation of HyperBlend wrongly account for photons that do not scatter in the light port of the integrating sphere (or its virtual counterpart) as absorbed, which overestimates the particle density of the virtual material. In the validation experiment simulation, this was corrected by visually matching the scattering pattern of the virtual microalgae solution to match the light cone of the real-world scene. The correction was done through dilution of the virtual suspension by multiplying the densities of scattering and absorbing particles given by the slab simulation with a Lagrange multiplier of 1/4. Approximate similarity can be verified by comparing figure 8*a*,*b*. This was further verified by inspecting the rate at which the upward scattering decays as the optical path in the algae suspension becomes longer. A more detailed analysis of this can be found in §3.1.

### 2.4. Photobioreactor design experiment

For testing a few different reactor design choices, we consider photobioreactors that are geometrically right circular cylinders with a top (lid) and a bottom. To showcase the illumination problem, we use three different reactor volumes ranging from a laboratory-sized 10 l reactor to a prototype industrial scale of 1000 l and a 100 l reactor as a stepping stone. The geometry was locked down so that the height of the photobioreactor is two times its radius h=2r and its volume is therefore $V=2\pi r^{3}$. With this selection, the geometry is now bound to the volume because for any V we can calculate the radius $r=\sqrt[{3}]{V/2\pi }$. We do not suggest that this is the optimal geometry for a cylindrical photobioreactor but rather an arbitrary selection to allow comparison between different volumetric scales.

We consider two different reactor materials and lighting schemes necessitated by the materials: one is made of glass and is lit from the outside, through the wall of the reactor. One can think of a sheet of LED lights directly against the wall of the photobioreactor. The second is made of steel and is lit by placing rod-like lamps in circle(s) inside the reactor. In both cases, the reactor is considered to be filled to the brim with microalgae suspension and the lamp extends all the way from top to bottom.

For the glass photobioreactor, the inner volume is directly the volume of algae suspension that can fit inside. However, in the steel photobioreactor, the rod lamps inside the photobioreactor reduce the volume available for the algae suspension. We compensate for this by increasing the photobioreactor dimensions so that the working volume available for the solution is the same in both photobioreactor types.

For the steel photobioreactor, we use a certain number of rod lamps of a certain radius to produce the same lighting surface area as in the glass photobioreactor case for comparable results. As both photobioreactor types have the same lamp surface area $A_{l}$, we can set matching light power in watts per square metre. The dimensions for the glass photobioreactor are listed in table 2. For the rod lamps in the steel photobioreactor, we must decide the number of the lamps n and their radius $r_{l}$. We want the total lamp surface area to match the glass photobioreactor’s wall area so that the number of photons emitted from both light types can be matched by simply setting the same wattage per square metre. This is an arbitrary selection as we could also disregard the surface area and use lamp power to compensate.

**Table 2.** Glass photobioreactor dimensions and lamp areas.

volume (l)	r	h (cm)	$A_{l}$ $\left(m^{2}\right)$
10	11.68	23.36	0.1714
100	25.15	50.30	0.7949
1000	54.19	108.38	3.6902

Matching the working volume of the two reactor types is not quite as simple as it may seem at first glance. As we put some lamp rods into the steel photobioreactor, we decrease the working volume, which must be compensated for by increasing the outer dimensions of the photobioreactor. We have locked the photobioreactor geometry so that the height of the photobioreactor is two times its radius, and the lamp rods are as high as the inside of the photobioreactor. Therefore, by increasing the photobioreactor volume, we also increase the volume taken up by the lamp rod because it is now taller. This, in turn, has to be compensated by increasing the photobioreactor volume again. This leads to a nonlinear system of two equations and three unknowns, which becomes solvable if we lock one of the unknowns. The solution is non-trivial, and it is presented in appendix A. Table 3 lists the dimensions of the steel reactors after solving the system.

**Table 3.** Steel photobioreactors' dimensions, number of lamps, lamp radii and lamp surface areas.

volume (l)	r (cm)	h (cm)	n	$r_{l}$ (cm)	$A_{l}$ $\left(m^{2}\right)$
10	12.27	24.54	6	1.85	0.1714
100	25.81	51.63	12	2.04	0.7949
1000	54.82	109.64	28	1.91	3.6902

Now, we have three volumes, two reactor types and five microalgae samples resulting in 30 possible scenarios to simulate. We reduced the number of scenes by simulating all reactor types and volumes with algae sample 4, which has the closest cell count to the culture used in the validation experiment and can thus be expected to reflect the real world the best. To depict how the light attenuates in a more mature and dense culture, we also simulated sample number 1. All simulations used the same AP67 (Valoya) grow light spectrum used in the validation experiment. The light power was scaled so that the number of overexposed pixels was minimal while still retaining sufficient illumination in all test cases. The reactors were virtually imaged from above with the reactor lid removed. The density of the absorbing and scattering particles was in all cases multiplied by 1/4 as in the validation experiment.

As we are mainly interested in the absorption of wavelengths used in photosynthesis, and especially how the peak emission wavelengths of the grow light decay while traversing the microalgae culture, the simulation was run with 10 nm spectral resolution from 400 to 700 nm, resulting in 31 simulated bands per image cube as in the validation experiment. The execution time for each band rendered with 5000 light rays per pixel was around 20 s per band and 10 min for the whole image cube. Image rendering was run on a GeForce RTX 4090 (NVIDIA) graphics card using Blender v. 4.0.

## 3. Results

The spectra in this section are not normalized with the lamp spectrum and, as such, include the characteristics of the lamp. This highlights how the emission peaks of the lamp become absorption features when the light interacts with the microalgae suspension in the real world and the virtual world.

### 3.1. Validation experiment

**Figure 10.**
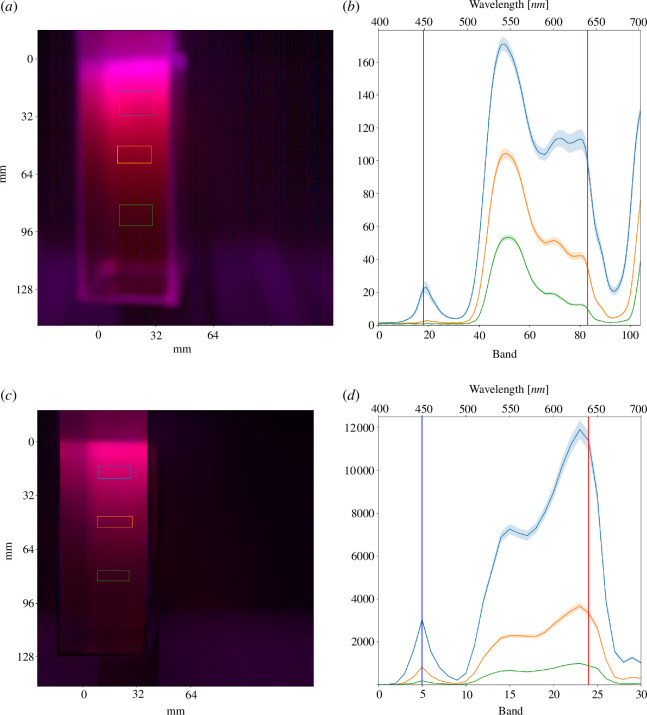
The false colour representation of the imaged (top row) and simulated (bottom row) hyperspectral cubes (left column) is constructed so that the blue channel is occupied by a 450 nm band and the red channel with a 640 nm band while the green channel is zeroed. Corresponding bands are shown in the mean spectra plots (right column) by blue and red vertical lines. Shadow plots show ± half of the standard deviation. The *y*-axis for spectra is arbitrary. Mean spectra regions are manually selected from the image cube as shown by the correspondingly coloured rectangles. Distances shown in false colour cubes are approximate and apply to the level of suspension.

Figure 10 shows the false colour presentation of the imaged and simulated spectral cubes where the blue channel uses values of the band closest to 450 nm and the red channel the one closest to 640 nm while the green channel is zeroed out. Including the green channel would provide a more natural look, but as the green light is not very active photosynthetically, its inclusion would give the wrong idea of how well the light penetrates the suspension. In other words, as the green light is not absorbed by the algae as actively as blue and red, it can penetrate much deeper into the suspension, but the penetration ability of the green light is not interesting as it is not important for the algae. The false colour presentations in figure 10 closely resemble each other in geometry and light scattering, though they cannot be compared on a pixel-by-pixel level. The *x*- and *y*-axes of the false colour cubes show approximate distance at the level of the suspension, and the origin is placed in the top left corner of the cultivation bottle.

A few representative regions were selected from both image cubes, and their spectra are plotted in the right column of figure 10. There is a distinct difference in the shape of the spectra between the imaged spectral cube (top row) and the simulated cube (bottom). The greatest disagreement is in the green region around 550 nm where the green peak has been absorbed away in the simulation. We suspect that this is due to the earlier mentioned problem where scattering is attributed to absorption in the simulation. This does not happen in the actual laboratory set-up, of course, as the green light is free to scatter. Regardless of the erroneous simulation result on the green, the blue and the red bands (indicated by vertical lines at 450 and 640 nm) show good agreement with the real-world measurement. The height of the blue peak compared with the height of the red peak is proportionate. In addition, the scattering intensity while traversing further to the solution decays similarly in both.

**Figure 11.**
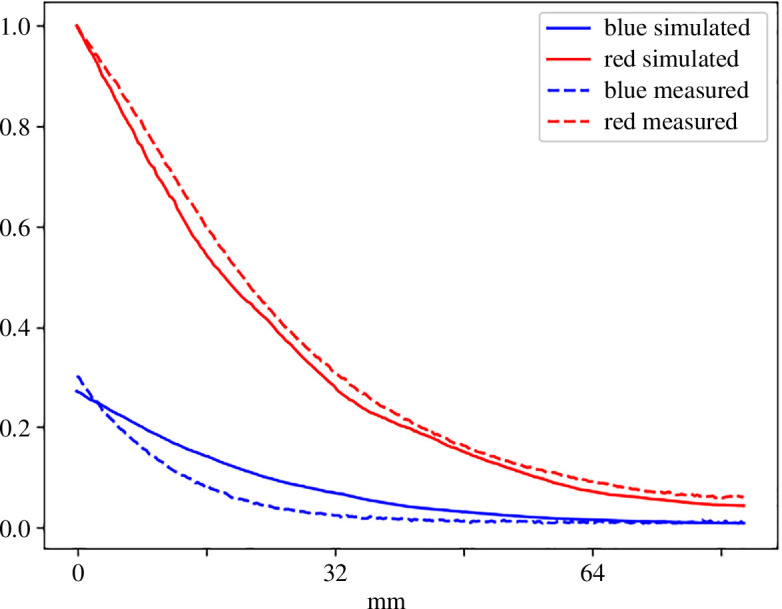
Simulated and measured decay of light scattered upwards as a function of distance traversed in algal culture. Shown values are mean values approximately from the width of the cultivation bottle. Both are normalized so that the brightest pixel on the red band equals unity. Note that the zero of the *x*-axis does not match the origin of false colour cubes in figure 10.

The penetration of light at the emission peaks of the lamp, in our case indicated by upward scattering, is worth further inspection as it tells whether or not the simulation is usable in assessing light conditions inside a larger photobioreactor. Figure 11 shows the decay of 450 nm and 640 nm in the measured (dashed lines) and the simulated (solid lines) spectral cubes. Plotted lines show the mean (*x*-direction) upward scattered intensity starting from the brightest part at the top of the cube in figure 10*a*,*c* going downwards for about 83 mm until the edge of the bottle. Both are normalized so that the brightest pixel value (on the red band) equals unity. The simulated red band is in good agreement with the imaged cube, while the blue band decays slightly too slowly. The optical path length of the culture bottle in this set-up is roughly 110 mm, so by this result, we can expect the simulation to be fairly accurate, at least for this distance and cell density. The density of the algae suspension also affects how fast the simulated light rays die off, so no definite limit to the light path length for accurate simulation results can be given.

The virtual scene of the validation experiment is very sensitive to small changes in the scene geometry. Even a few millimetre differences in the vertical positioning of the light source, for example, causes significant changes in the result. Some errors are likely due to the virtual scene not fully representing the actual laboratory set-up. The absorption of light into the virtual algae suspension is naturally also affected by the selection of the Lagrange multiplier used for the particle densities. Artificially diluting the virtual cell culture like this compensates for the imperfections in the simulation, but any selection is a trade-off where the shape of the decay may improve in some regions and for one of the bands but deteriorate somewhere else. Unfortunately, we are not able to provide proper error estimates of the simulation errors as there is a myriad of error sources of unknown quantity, beginning from the initial spectrophotometer measurements. Regardless of these clear deviations from the real-world scenario, we deem that the simulation can approximate light penetration into algae suspension accurately enough to be useful in photobioreactor design.

### 3.2. Photobioreactor design experiment

**Figure 12.**
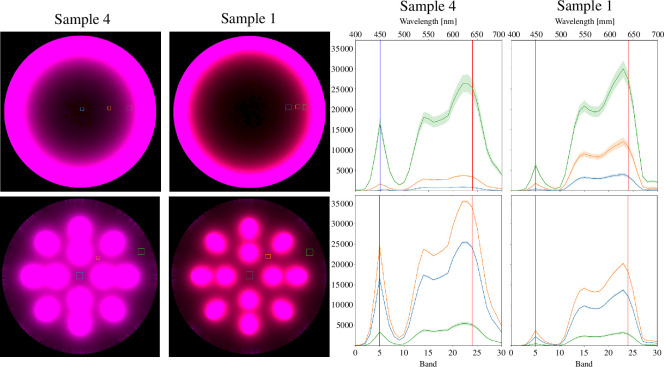
False colour images of simulated spectral cubes of the 100 l glass (top row) and the 100 l steel (bottom row) photobioreactors and their respective spectra on selected regions (indicated by colour-coded rectangles). Columns indicate which measured microalgae spectrum was used in the simulation: sample 1 is the densest suspension, and sample 4 is roughly as dense as the one used in the validation experiment.

False colour presentations of the simulated 100 l glass and steel photobioreactors are shown in figure 12 for algae samples 1 and 4 (see table 1). We selected the 100 l reactor for closer inspection as it shows the light dissipation most clearly. The 10 l reactor is so small that both reactor types can light it properly. The 1000 l reactor, on the other hand, is perhaps an overly naive example as its radius is over half a metre, and the light penetrates very poorly for such a long distance, regardless of the microalgae cell densities used in the experiment. The result images of the 10 l and the 1000 l reactors are shown as electronic supplementary material in appendix B. Regions where the mean spectra are plotted were selected manually from the area where the most interesting light interactions happen: for the glass reactor, it is the region where the light intensity begins to visibly fade, and for the steel reactor, the darkest regions between the lamps and close to the wall of the reactor.

The magnitude of the plotted spectra of the design experiment has no physical meaning, but they can be compared against each other as the power per lamp surface area in all simulations is the same. The plotted spectra of the steel reactor in the bottom row of figure 12 show that even when the cell density increases fivefold from sample 4 to sample 1, the light intensity is still good throughout the reactor. The spectra of the glass reactor in the top row do not show great change, which is because the regions where the mean pixel value has been plotted are considerably closer to the glass wall.

All simulated images of the steel reactors show a specular reflection artefact as bright spikes along the wall of the reactor. The artefacts do not show when a single band is rendered directly from Blender’s graphical user interface (GUI), so we suspect that there might be differences in how Blender handles render calls coming through its Python API compared with using the GUI. These particular artefacts could result if interpolation of the vertex normals is not applied either for the rod lamps or the steel wall.

The false colour images of the denser sample 1 in figure 12 show a clear red halo at the edge of the well-lit area where the blue wavelengths zero out before the red, even though the blue absorption is underestimated in the simulation as shown by the validation experiment. The blue emission peak of the lamp is stronger than the red one (as seen in figure 9), but also the absorption is stronger in the blue as shown by the spectrophotometer measurements (figure 4), so the simulation behaves as expected. This is further evidenced by the spectra of selected image regions where the relative height of the blue peak is significantly lower in the spectra of sample 1 than in the spectra of sample 4. As the density of the microalgae culture increases, the absorption rate of blue light increases faster than the absorption rate of red light. The spectra of both samples also show the overestimation of green absorption as expected according to the results of the validation experiment.

It is worth noting that in the 100 l glass reactor, for the more dilute sample 4, the signal is not lost even at the very middle of the reactor. In the denser sample 1, the regions are selected slightly outside of where the blue peak vanishes. As the radius of the reactor is about 25 cm, the cells further than roughly 10 cm from the wall do not receive blue light at all. In the steel reactor, there is no location completely lacking blue light.

The standard deviation of the plotted spectra in figure 12 shows that the light field is much more even in the steel reactor. At any point in the reactor, the distance from the light source is short and the reflective steel wall evens out the light field. In the glass reactor, the rapid change in the light field is especially evident close to the wall. While the evenness of the light may not be a virtue in itself, a highly heterogeneous light field requires rigorous mixing of the suspension so that all cells can have enough light for photosynthesis at least part of the time.

For a quantitative comparison between all reactor types and selected samples on the blue and red channels, we calculated all pixels where the intensity exceeds 1000. A binary image mask was rendered from Blender, so that only the pixels at the surface of the suspension are included and lamps, reactor walls and the background are excluded. The number of pixels in this region of interest that exceed the selected threshold value is divided by the number of all pixels in the region of interest.

The percentages of the pixels exceeding the threshold are listed in table 4. It is clear that regardless of suspension density, the steel reactor has a considerably higher percentage of ‘well-lit’ pixels than the glass reactor does. The 10 l reactors do not show many differences, as they are essentially fully lit regardless of the reactor type. The differences between the steel and the glass reactor types are clear in favour of the steel reactor in the 100 and 1000 l reactors. Where there are differences between the blue and red channels, the red has, as expected, a consistently higher percentage of well-lit pixels.

**Table 4.** Percentages of pixels exceeding a threshold value in all reactor types, volumes and selected samples for the blue (B columns) and the red channel (R columns). Sample 1 is the densest suspension, and sample 4 is roughly as dense as the one used in the validation experiment.

	sample 4		sample 1	
	B (%)	R (%)	B (%)	R (%)
glass 10 l	100.0	100.0	96.7	100.0
steel 10 l	100.0	100.0	100.0	100.0
glass 100 l	89.4	97.0	62.4	85.3
steel 100 l	100.0	100.0	71.4	99.9
glass 1000 l	52.5	59.1	34.2	49.5
steel 1000 l	83.3	91.1	48.7	79.5

## 4. Discussion and conclusions

The availability of illumination is a key problem in scaling photobioreactors to industrial volumetric scale [11]. Computer simulations are becoming important tools that facilitate the estimation of light availability in different photobioreactor designs. In the boreal and arctic zones, the placement of photobioreactors indoors and the provision of adequate lighting are key to reactor design. However, outdoor cultivations could also benefit from the optimization of light conditions through simulations. Evenly distributed irradiance adjusted to the attenuation by microalgae in the suspension is of crucial importance in ensuring controlled autotrophic growth. The locations of the dark and light regions, which are central to optical photobioreactor modelling [25], could be observed from the HyperBlend simulations. A metal cylinder with cultivation lamps placed inside the reactor provided notably more even light for growing *Chlorella vulgaris* compared with a glass photobioreactor illuminated outside the reactor. These results are in line with [3]. Although light distribution was superior in internally illuminated cultures, technical difficulties such as possible heat transfer from light sources to cultivation media and the formation of biofilms around the light sources are aspects that need to be considered in the design of an optimal cultivation setup.

3D modelling combined with ray tracing is a powerful tool for simulating realistic light interactions in photobioreactor design. Especially procedural modelling allows rapid prototyping of different configurations and volumetric scales. Conventional 3D modelling is still useful when testing more complex shapes or when creating digital twins of existing systems. Our simulation scheme allows the geometry and light spectrum to be adjusted in a very flexible manner, but it might be beneficial to couple it with CFD modelling in the future, for example, for locating possible clumping inside the reactor due to the flow of the suspension. CFD modelling could also be used in investigating gas–liquid mass transfer and its effect on algal biomass production [8]. The actual development work needed for coupling HyperBlend to a CFD model might not be straightforward, due to Blender’s limitations in defining heterogeneous density inside a volumetric shader. Even though the RT-similarity requirement was greatly alleviated in this study, it is still present. Especially if, in the future, one would like to simulate a situation where reflectance is considerably higher than transmittance, the internal approximation methods of HyperBlend must be retrained.

We do not propose that the photobioreactor designs presented in this study are optimal for cultivating microalgae in general. Instead, we highlight the ease of experimenting with different reactor designs in the virtual environment before committing to manufacture a fully working physical prototype. Although we only tested simple cylindrical photobioreactor shapes in this study, the developed approach is fully capable of simulating flat-panel and tubular photobioreactors of any shape and complexity. Utilizing HyperBlend allows us to duplicate real microalgae cultures in the virtual world through their spectral transmittance and reflectance. In future studies, we should consider building a model, perhaps a neural network, to connect reflectance and transmittance spectra to the density of the algae suspension, which would facilitate the estimation of light propagation in a growing cell culture where the density of the suspension is constantly changing. That kind of simulation could be used to estimate the sufficient increase in light intensity as the culture matures.

Spectral simulation accuracy in this study was hindered by attributing scattering events to absorption. This led the simulation to overestimate absorption in the whole visible wavelength region and the simulator had to be manually adjusted by introducing a Lagrange multiplier that effectively diluted the density of the virtual culture to one-fourth of the original density. The overestimation was not constant on the whole wavelength region, as even with the adjustment, the absorption of green wavelengths was still too high. We suspect that the disproportionate simulation error in the green is associated with the low absorption and high scattering features of the green light in microalgae suspensions which should be considered in the future, both when measuring the spectral properties of the real-world algae, and also in the implementation of the simulator. Regardless, the absorption at the most important chlorophyll absorption features at 450 and 640 nm were accurate enough to be used for estimating the penetration of photosynthetically active radiation into the microalgae culture.

Judek *et al*. [22] propose calculating the scattered component of incident light by measuring a cuvette filled with suspension inside an integrating sphere in addition to front and back port measurements. They acknowledge that the measurement is prone to error, as even small deviations in the positioning angle of the cuvette affect the measurement. In addition, this method is risky for the measuring equipment itself, as spilling the contents of the cuvette inside the integrating sphere would likely damage it, perhaps permanently. As the cost of an integrating sphere is several thousand euros, the risk cannot be neglected. One considerable option would be to use less expensive, and thus more disposable, self-made integrating spheres such as the one suggested by [26]. In addition to measuring the scattered light, implementing more robust scattering control to the HyperBlend simulation would require considerable development work as the most fundamental core assumptions of the simulator would have to be rewritten. Possible solutions could be using more camera angles to cover all the sides of the slab in the slab simulation phase or 3D modelling a virtual integrating sphere to better match the real-world measurement setup. These possibilities should be explored in future studies for more robust simulations.

The software code used for this study was drastically modified from the original source code of HyperBlend. For that reason, it has been made available as a separate Git fork at [27]. In the future, the main fork of HyperBlend should be refactored so that it can accommodate more varied needs for spectral simulations without requiring rewriting of substantial portions of the code.

## Figures and Tables

**Figure 13.**
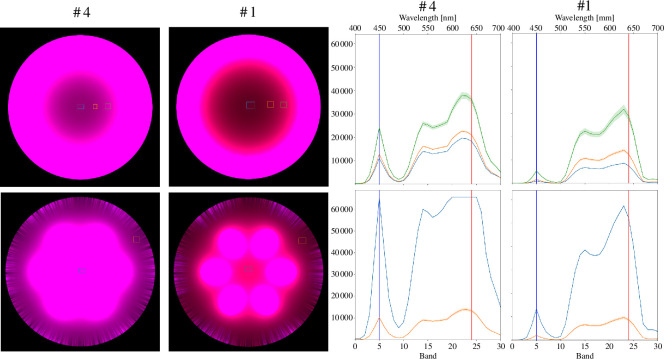
False colour images of simulated spectral cubes of the 10 l glass (top row) and the 10 l steel (bottom row) photobioreactors and their respective spectra on selected regions (indicated by colour-coded rectangles). Columns indicate which measured microalgae spectrum was used in the simulation: sample 1 is the densest suspension and sample 4 is roughly as dense as the one used in the validation experiment.

**Figure 14.**
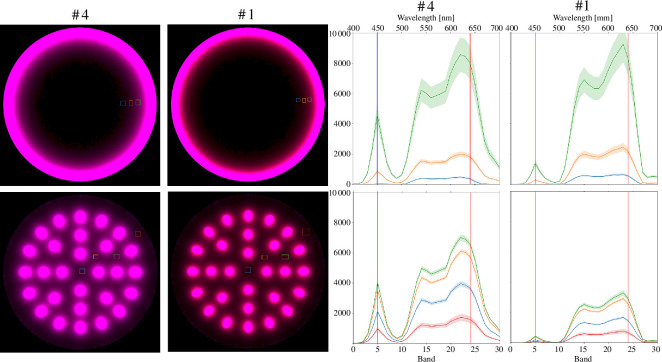
False colour images of simulated spectral cubes of the 1000 l glass (top row) and the 1000 l steel (bottom row) photobioreactors similarly to the previous figure.

## Data Availability

The software code of the simulator is available at [27]. The repository also contains spectrophotometer measurements of the *Chlorella vulgaris* samples used in this study. A permanently stored version of the code can be found at [28]. It also contains the simulated hyperspectral image cubes that are not stored in the GitHub repository. The source code of the CubeInspector software used to inspect the ENVI-formatted spectral cubes in this study is available at [29]. CubeInspector was used to create all the spectrum plots and false colour presentations of the spectral cubes in this article. A frozen version of the code can be accessed at [30].

## Appendix A: Calculating the steel photobioreactor lamp size

In this study, the glass photobioreactor is geometrically a cylinder whose height is twice its radius. The steel photobioreactor is similarly a cylinder but also has cylindrical lamps placed inside it that take up some space, which must be compensated by increasing its dimensions, which, in turn, increases the length of the lamps. This leads to a system of two equations with three unknown variables that can be solved by fixing one of the variables as follows. Subscript *g* indicates variables related to the glass reactor and *s* to the steel reactor, respectively. For simplicity, we assume the wall of the reactor is thin, and its effect on the surface area of the glass reactor’s lamp is neglected.

Let the working volume of the glass photobioreactor be

(A 1)
$$V_{g}=2\pi r_{g}^{3},$$

and its wall’s surface area $A_{g}$ that is used as a light source

(A 2)
$$A_{g}=4\pi r_{g}^{2},$$

where we use the relation $h_{g}=2r_{g}$.

We now want to build a steel photobioreactor whose dimensions must be bigger because we put some rod lamps inside that decrease the working volume. The total volume of the steel photobioreactor is similarly

(A 3)
$$V_{s}=2\pi r_{s}^{3}.$$

The total volume taken up by all of the lamp rods is

(A 4)
$$V_{l}=2n\pi r_{l}^{2}r_{s},$$

where n is the number of lamps, $r_{l}$ is the radius of a single lamp and $2r_{s}$ is still the height of the steel photobioreactor. The total lighting area of the lamp rods is

(A 5)
$$A_{l}=4n\pi r_{l}r_{s}.$$

We established two conditions for the steel photobioreactor: the total lamp area and the working volume of both photobioreactor types must be the same, which leads us to a system of equations

(A 6)
$$\left\{ \begin{array}{l} V_{s}=V_{g}+V_{l}\\ A_{l}=A_{g} \end{array}\right..$$

Substituting equations (A 1)–(A 5) into equation (A 6) yields

(A 7)
$$\left\{ \begin{array}{l} 2\pi r_{s}^{3}=2\pi r_{g}^{3}+2n\pi r_{l}^{2}r_{s}\\ 4n\pi r_{l}r_{s}=4\pi r_{g}^{2} \end{array}\right..$$

Now, we have three unknowns $r_{s}$, $r_{l}$, and n but only two equations, so this system of equations cannot be solved. However, we can guess a value for one of the unknowns and pretend we know it beforehand. This will allow us to solve the problem. The easiest unknown to be locked down is the number of lamps n. We can say we want to use four lamp rods, for example, and now the system can be solved. The radius of the lamp rods and the radius of the steel photobioreactor will adjust accordingly. If the guess provides an unreasonably small or big lamp radius, we can just select a different n.

Solving and simplifying the lower equation of equation (A 7) for lamp radius yields

(A 8)
$$r_{l}=\frac{r_{g}^{2}}{r_{s}n}.$$

Substituting that back into the first equation of equation (A 7) and simplifying it all down yields

(A.9)
$$\left\{ \begin{array}{l} r_{s}^{3}=r_{g}^{3}+\frac{r_{g}^{4}}{r_{s}n}\\ r_{l}=\frac{r_{g}^{2}}{r_{s}n} \end{array}\right..$$

The first equation is now a third-order polynomial $r_{g}^{3}-r_{s}^{3}+\frac{r_{g}^{4}}{r_{s}n}=0$, which can be solved for $r_{s}$ using a symbolic calculator with a selected value for n. The result can then be substituted into the second equation and the system is solved.

## Appendix B: Simulation results of the 10 and 1000 l reactors

Simulation results for 10 and 1000 l reactors are shown in figures 13 and 14, respectively. The 10 l reactor is well-lit regardless of the density of the suspension and the reactor type in the sense that the light signal does not die off completely on any band. The spectrum of the more dilute sample 4 in the steel reactor (bottom row, second image from right) is clipping at the maximum value of 65 535 of the 16-bit unsigned integer used in saving the rendered images.

As expected, the false colour images of the 1000 l reactor in figure 14 clearly show that building a glass reactor of that size is not beneficial as light can only penetrate a small fraction of its half a metre radius. The 1000 l steel reactor is evenly lit, but the intensity of the light is fairly low.

The *y*-axis of the plotted spectra does not have any physical meaning, but they allow comparing the spectra in this study against each other. In the 10 l reactors in figure 13, the spectra of the glass reactor maxes out between 30 000 and 40 000, while in the steel reactor the maximum peak values are well over 60 000. Even while both reactors have plenty of light, the steel reactor could reach the same light intensity with lower lamp power, as the lamps in both reactors use the same total amount of power. A similar comparison between the two reactor types in the 1000 l configuration shows that even if the maximum peaks of the spectra of the glass reactor are higher at about 10 000 compared with the steel reactor maximum peaks between 3000 and 8000, the regions of interest in the glass reactor are close to the wall, while the selected regions of the steel reactor contain the darkest areas that can be found.
